# Weighted Matrix Decomposition for Small Surface Defect Detection

**DOI:** 10.3390/mi14010092

**Published:** 2022-12-29

**Authors:** Zhiyan Zhong, Hongxin Wang, Dan Xiang

**Affiliations:** 1 School of Automation, Guangdong Polytechnic Normal University, Guangzhou 510665, China; 2 Machine Life and Intelligence Research Center, Guangzhou University, Guangzhou 510006, China; 3 School of Computer Science, University of Lincoln, Lincoln LN6 7TS, UK; 4 Guangzhou Maritime University, Guangzhou 510725, China

**Keywords:** defect detection, machine vision, weighted matrix decomposition

## Abstract

Detecting small defects against a complex surface is highly challenging but crucial to ensure product quality in industry sectors. However, in the detection performance of existing methods, there remains a huge gap in the localization and segmentation of small defects with limited sizes and extremely weak feature representation. To address the above issue, this paper presents a weighted matrix decomposition model (WMD) for small defect detection against a complex surface. Firstly, a weighted matrix is constructed based on texture characteristics of RGB channels in the defect image, which aims to improve contrast between defects and the background. Based on the sparse and low-rank characteristics of small defects, the weighted matrix is then decomposed into low-rank and sparse matrices corresponding to the redundant background and defect areas, respectively. Finally, an automatic threshold segmentation method is used to obtain the optimal threshold and accurately segment the defect areas and their edges in the sparse matrix. The experimental results show that the proposed model outperforms state-of-the-art methods under various quantitative evaluation metrics and has broad industrial application prospects.

## 1. Introduction

Under the trend of intelligent manufacturing, automatic quality control is widely regarded as the top priority in industrial production [1,2]. Surface defect, the key factor of quality control, is generally defined as local anomalies embedded in homogeneous textures [3]. Due to complexity of manufacturing processes and diversity of production environments, defects on product surfaces are always various and complex. For example, the manufacturing process of powder metallurgy is composed of casting, forging, rolling, machining, and extrusion, while its porosity, an important characteristic of powder metallurgy sintered materials, is influenced by multiple factors including temperature, sintering time, and pressure [4]. Surface defects not only directly lower appearance quality, but also reduce product performance and commercial value [5]. To effectively detect surface defects, automatic visual inspection methods with great advantage in non-destructive defect detection have been widely applied in rails [6], fabric [7], steel [8], thin-film-transistor [9], photovoltaic [10], and other flat products [11].

Conventional defect detection methods can be roughly divided into three categories: statistical-, spectral-, and model-based techniques [3]. Statistical-based approaches compute local features at each pixel and then derives a set of statistics from distributions of the local features to discriminate defects [12]. They generally contain eight representative algorithms, including thresholding [13], clustering [14], edge-based [15], fractal dimension [16], gray-level statistic [17], co-occurrence matrix [18], local binary pattern [19], and morphological [20]. Among them, the most classical method is thresholding. Ostu presented a threshold selection method from gray level histograms, which is optimal for thresholding a histogram with bimodal or multimodal distribution but fails if the histogram is unimodal or close to unimodal [21]. To address this issue, a number of modified methods were developed based on the work of Ostu [22]. For example, Ng et al. proposed the valley-emphasis method (VE), which is suitable for images with unimodal and bimodal distributions [23]. To obtain better and more stable thresholding results, the Gaussian valley-emphasis method (GVE) was developed [24]. Truong et al. designed an entropy weighting scheme (EnOstu) to improve upon Otsu’s method [25]. In addition, neighborhood valley-emphasis method (NVE) [26] and improved valley-emphasis method (IVE) [27] were proposed for small surface defect detection. Statistical-based approaches perform well on defect detection against steel surface whose pixel intensities exhibit remarkable regularity and apparent periodicity. However, they are vulnerable to illumination change [28], pseudo defects, and local noise [29].

To overcome the above limitation, the second method transforms the whole image from spatial domain to frequency domain where defects can be discriminated from defect-free regions more easily via comparison of their amplitudes [3]. Spectral-based approaches are composed of six representative algorithms, such as Fourier transform [30], Gabor filters [31], optimized FIR filters [32], wavelet transform [33], multiscale geometric analysis [34], and hough transform [35]. Tsai et al. proposed fabrics defects inspection method based on a global image restoration scheme using the Fourier transform, which does not rely on local features of textures [36]. Similarly, Hu et al. combined Fourier analysis and wavelet shrinkage to detect textile defects [37]. Aiger et al. presented a novel method based on the Phase Only Transform (PHOT) [38] for detecting defects on textured surfaces. Its simplicity and generality enable it to work on various pattern without prior knowledge. Choi et al. proposed an unsupervised detection approach of surface defects by combining both global estimation and local refinement, which gives robust results even in noisy surface defect images [39]. However, the spectral-based method is unable to effectively preserve local information after the transformation, which means it can only deal with defects that occupy a large amount of the whole image and exhibit significant difference to the background.

To achieve better performance for diverse defects, the third method projects original texture distribution of image blocks into low-dimensional space using different specialized models [40], such as Markov random field [41], Gaussian mixture entropy [42], and Weibull distribution [43]. Model-based approaches accomplish defect detection by similarity measurement between the model which is established by the feature of image and the test image [44]. Susanet et al. proposed a Gaussian mixture entropy model to automatically defect detection with no manual intervention [42]. Han et al. developed a novel methodology for anomaly detection in noisy images with smooth backgrounds, named smooth-sparse decomposition (SSD) [45], which has superiority in terms of the detection accuracy as well as computation time. The following year, they proposed a spatio-temporal smooth sparse decomposition [46], which has the capability of identifying not only the time of process changes, but also the location of detected anomalies. Yu et al. proposed a coarse-to-fine model to identify rail defects at different scales [47]. However, the model-based methods are designed for specific defects and difficult to generalize for various types of defects [48].

Most of the aforementioned approaches distinguish defects from background by extracting the textural features [8]. The statistical and spectral methods can hardly deal with the situation when detect exhibit low contrast against complex background [49]. The model-based approaches tend to consider the feature of the test images, which means the model is always specific and difficult to achieve universality. Convolutional neural networks (CNNs) have become a key element in the breakthrough of defect detection [50,51,52], but they may fail to detect small defects that are only a few pixels in size, since rich representations are difficult to learn from their poor-quality appearance and structure. Another obstacle presented by convolutional neural networks is that downsampling of the image always misses the opportunity to locate small defects; for example, YOLO apply a downsampling of 32×32 to the input image, which means a defect with only a few pixels will disappear in the feature map [53]. In view of these points, the above conventional methods are difficult to be applied directly for the inspection of small defects on surfaces with complex backgrounds. Although these conventional defect detection methods have accomplished grate success in the detection of specific defects, their performance still needs to be improved for small defects with extremely low gradient intensity or contrast to background while equate to only a few pixels in size within images.

**Figure 1:**
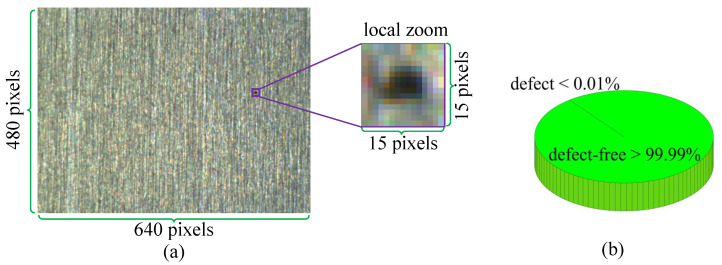
Examples of small surface defect dataset [54]. (**a**) Typical challenging examples of small surface defect. (**b**) The ratio is calculated over the defective or defect-free regions to an image.

Detecting small defects against complex surfaces has been an extremely challenging task for industrial manufacturing. Figure 1 gives an example of small surface defect [54]. As shown, the resolution of the whole image is 640×480 pixels where the defect and its surrounding region are enlarged in a square box with 15×15 pixels in size. Figure 1b presents the ratio of the number of pixels in the defect region to the total pixel number of the original image. As can be seen, the proportion of the small defect to the whole image is quite low (less than 0.01%). In this case, it is difficult even for humans to notice such tiny defects. Specifically, difficulties for small defect detection are reflected in three aspects:

(1) Small defects always equate to a few pixels in size, revealing almost no other visual features, such as shape, texture, and color, which means their feature representations are extremely weak.

(2) Low contrast and unclear boundary between defect and defect-free regions of complex surface bring great uncertainties for defect feature extraction.

(3) Noise inevitably introduced in image acquisition processes is difficult to discriminate from small defects and also leads to inaccuracy of defect area segmentation.

To address these issues, a weighted matrix decomposition model (WMD) is proposed for small defect detection against complex surface. Specifically, a matrix is constructed by weighted summation of three channels from the original color image to improve the contrast between small defects and background. The weighted matrix is then decomposed into a sparse matrix and a low-rank matrix to identify defect regions while eliminating the interference of complex backgrounds. The pixel intensity of the defects identified by the sparse matrix are further analyszd. Finally, the binarization operation and the pixel value statistical analysis of the connected domain are performed to accurately segment defect regions and effectively filter out noise.

The rest of our paper is organized as follows. In Section 2, the weighted matrix decomposition model is introduced. In Section 3, experimental results on small surface defect detection are presented, together with the qualitative studies. Finally, the main idea and future work of this paper is summarized and concluded in Section 4.

## 2. Weighted Matrix Decomposition Model

### 2.1. Overall Network Architecture

**Figure 2:**
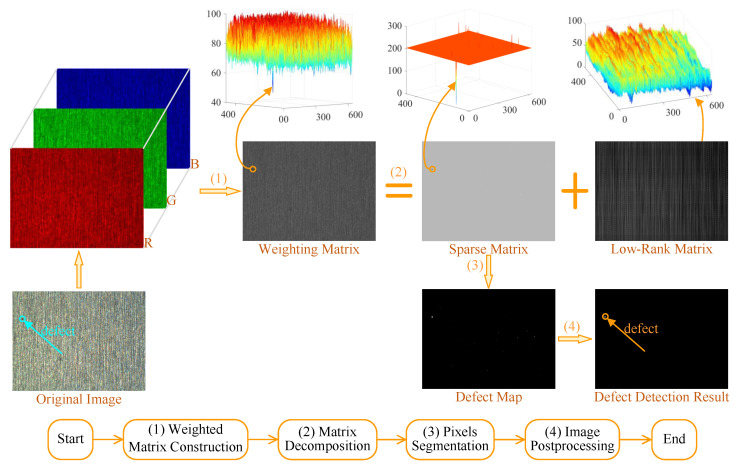
Framework of the WMD model for defect detection.

The total framework of the proposed weighted matrix decomposition (WMD) model is shown in Figure 2. As can be seen, it is composed of three main steps, including weighted matrix construction, image decomposition, and defect region segmentation. Specifically, a weighted matrix is firstly constructed for a color image to improve contrast between defect and defect-free regions. The weighted matrix is further decomposed into a low-rank and sparse matrices to eliminate redundant background. Finally, defective regions in the latter matrix are located by an adaptive threshold. The following sections will elaborate on each step of the proposed WMD model.

### 2.2. Weighted Matrix Construction

**Figure 3:**
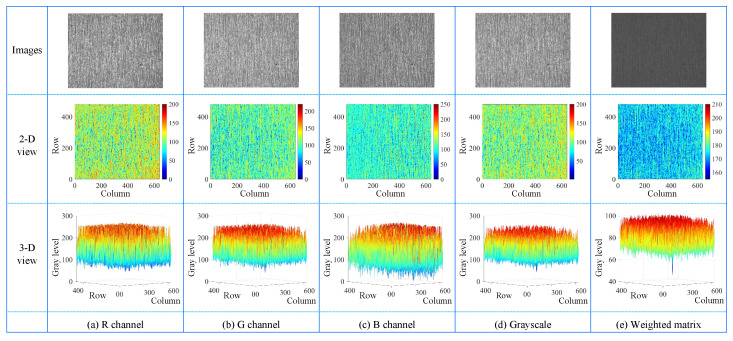
The first column shows R channel, G channel, B channel, grayscale version, and weighted matrix of a steel surface image, respectively, while the second and third columns present their corresponding 2D and 3D views. The weighted matrix is derived from the weighted summation of the R, G, and B channels.

Figure 3 shows the 2D views and 3D views of the RGB channels and gray-scale version of a steel surface image. As can be seen, pixel values of the defect are extremely close to those of the background. In this case, it is quite difficult to discriminate the defect and defect-free regions. To solve this problem, a weighted matrix is proposed to enhance contrast, which consists of three steps, including normalization, weighted summation, and linear transformation.

(1) Normalization

The pixel values [0,255] of the RGB channels are all normalized to [0,1] for the convenience of calculation, that is,

(1) I(x,y,z)=f(x,y,z)/255

where f(x,y,z) denotes input color image, (x,y) represents spatial coordinates, and z∈{1,2,3} is the channel index corresponding to the R, G, B channels, respectively.

(2) Weighted Summation

To closely match human perception of lightness, weights of the RGB channels are assigned based on the CIELAB color space [55]. Specifically, the higher contrast between defect and defect-free regions, the larger the weight. As shown in Figure 3, the contrast between the defect and defect-free regions in the three channels are arranged in descending order as G, R, and B channels. The weighted summation of the RGB channels is expressed as

(2) $$I_{M}\left(x,y\right)=u_{1}\cdot I\left(x,y,1\right)+u_{2}\cdot I\left(x,y,2\right)+u_{3}\cdot I\left(x,y,3\right)$$

where $u_{1}$, $u_{2}$, and $u_{3}$ are constant and satisfy $u_{2}>u_{1}>u_{3}$, $u_{1}+u_{2}+u_{3}=1$. To further enhance the contrast of the defect, the cube root of the weighted summation is adopted, that is

(3) $$I_{L}\left(x,y\right)=I_{M}{\left(x,y\right)}^{1/3}$$

where $I_{L}\left(x,y\right)$ denoted the contrast-enhanced matrix.

(3) Linear Transformation

The pixel values of $I_{L}\left(x,y\right)$ are linearly transformed to integer values from 0 to 255, that is

(4) $$I_{W}\left(x,y\right)\approx 255\times \frac{I_{L}\left(x,y\right)-min\left(I_{L}\left(x,y\right)\right)}{max\left(I_{L}\left(x,y\right)\right)-min\left(I_{L}\left(x,y\right)\right)}$$

where $I_{W}\left(x,y\right)$ is a positive integer matrix. If there are decimals in Equation (4), we round them up to the nearest integer. In this stage, we obtain the weighted matrix $I_{W}\left(x,y\right)$, where the contrast of the defect is significantly enhanced, as can be seen from Figure 3. $I_{W}\left(x,y\right)$ is further decomposed in the following sections to separate defects from the background.

### 2.3. Matrix Decomposition

A weighted matrix $I_{W}$ is first partitioned into *N* nonoverlapping patches, and then decomposed into sparse matrix $I_{S}$ with defects and low-rank matrix $I_{L}$ with redundant backgrounds. To address the issues discussed in Section 1, a novel weighted matrix decomposition model is proposed as follows:

(5) $$\begin{array}{c} I_{W}=I_{L}+I_{S} \end{array}$$

where $I_{L}$ is devoted to allow identification of the intrinsic feature subspace of the redundant background patches. $I_{S}$ is used to capture the spatial and feature relations of patches. Then, the defect detection task is transformed into a typical optimization problem of recovering low-rank and sparse components from a data matrix:

(6) $$\begin{array}{ccc} \; & \min_{I_{S},I_{L}}\;rank\left(I_{L}\right)+\lambda ||\left(I_{S}\right){\left|\right|}_{0}\; & s.t.\;I_{W}=I_{L}+I_{S} \end{array}$$

where the λ is weight coefficient (λ>0). *r* is a constant. $\left|\right|\cdot {\left|\right|}_{0}$ represents the $L_{0}$ norm to count the number of non-zero values. *k* is the number of pixels in the defect area.

**Figure 4:**
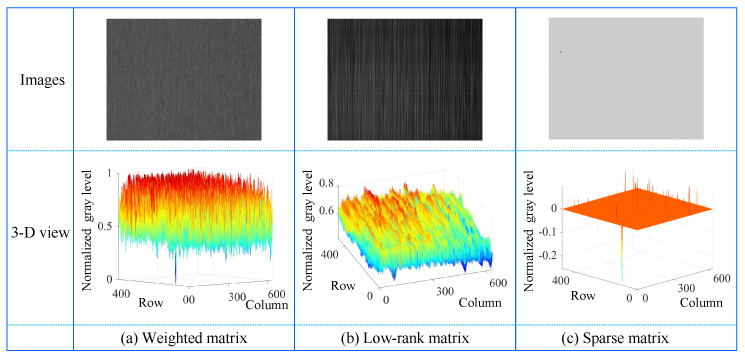
Matrix decomposition in single-defect images. The first column is a weighted matrix, which is decomposed into low-rank (in second column) and sparse matrices (in third column), respectively.

**Figure 5:**
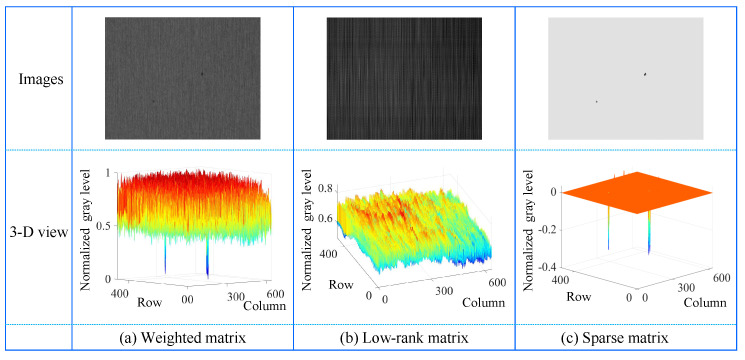
Matrix decomposition in multiple-defect images. The first column is a weighted matrix, which is decomposed into low-rank (in second column) and sparse matrices (in third column), respectively.

Figure 4 illustrate the matrix decomposition process for an image containing single defect, while Figure 5 present the matrix decomposition process for an image containing multiple defects. In each figure, the first column shows visual images of the weighted matrix, low-rank matrix, and sparse matrix after integerization while the second column presents their 3D views. In the following, the regularization of sparsity and low-rank matrices is verified, and their decomposition is implemented.

(1) Sparsity Regularization for Salient Defects

The contrast between defect and defect-free is glaringly obvious in the sparse matrices of Figure 4 and Figure 5. In their 3D view images, it is quite clear that most of the values are 0. Since the defect area is particularly smaller than the input image, the $I_{S}$ satisfies the following requirements:

(7) $$\left|\right|\left(I_{S}\right){\left|\right|}_{0}<k$$

where the image size is *M* × *N*, namely:

(8) k≪M×N

Equation (8) means that most of the elements in matrix $I_{S}$ are 0. That is, the assumption that the weight matrix of small surface defect images have sparsity, holds.

(2) Low-Rank Regularization for Image Background

**Figure 6:**
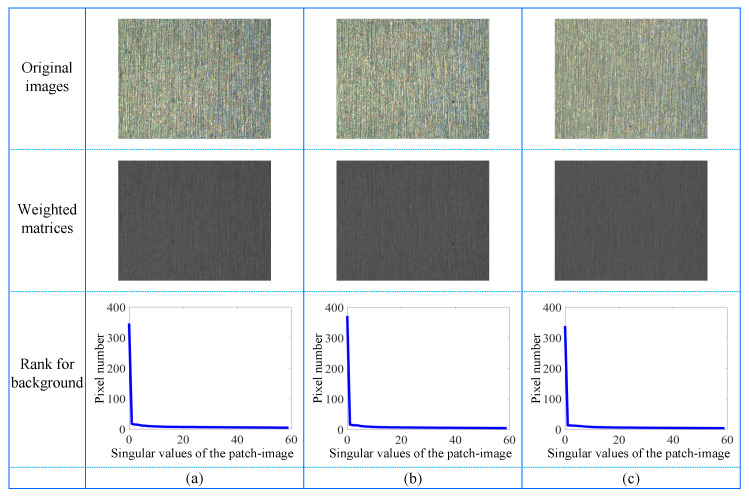
Low-rank matrix analysis. The first column shows three representative examples. In addition, the second and third one are weighted matrices and the singular values of the corresponding background patch-images.

As shown in Figure 4 and Figure 5, the texture of low-rank matrices is relatively smooth. If the image is divided into *L* blocks (where L<N), they are often similar and approximately lie in a low-dimensional subspace. The low-rank regularization on the background feature matrix $I_{L}$ is applied to pursue its intrinsic structure. Figure 6 presents three representative small surface defect images with the same size of 480×640×3, their corresponding weighted matrices in second column, and the singular value of the weighted matrix in third column where the patch size is 8×640 and the vertical sliding steps are 8. Since the sizes of all corresponding patch-images are 5120×60, the matrix has 60 singular values. The above analysis shows that the background feature matrix $I_{L}$ derived from the weighted matrix is low-rank, that is,

(9) $$rank(I_{L})\leq r$$

where *r* is a constant. Intrinsically, it constrains the complexity of the background image.

(3) Sparse and Low-Rank Matrices Decomposition

In order to detect small surface defect of images, the defect detection task is intrinsically a typical problem of recovering a low-rank and sparse components from a data matrix. This problem can be effectively solved via Principal Component Pursuit [56] and converted to solve the following convex optimization problem:

(10) $$\begin{array}{cc} \; & \min_{I_{S},I_{L}}\;||\left(I_{L}\right){\left|\right|}_{*}+\lambda ||\left(I_{S}\right){\left|\right|}_{1}\\ \; & s.t.\;I_{W}=I_{L}+I_{S} \end{array}$$

where λ is a positive constant. $\left|\right|\left(I_{L}\right){\left|\right|}_{*}$, $\left|\right|\left(I_{S}\right){\left|\right|}_{1}$ replace $rank(I_{L})$ in Equation (9) and $\left|\right|\left(I_{S}\right){\left|\right|}_{0}$ in Equation (7), respectively, for tractable computation. Here, $\left|\right|\cdot {\left|\right|}_{*}$ is the nuclear norm of a matrix (i.e., the sum of singular values); $\left|\right|\cdot {\left|\right|}_{1}$ is the $L_{1}$-norm, that is

(11) $${\left||X|\right|}_{1}=\sum_{ij}\left|X_{ij}\right|$$

The above optimization problem Equation (10) is convex and can be solved by applying the Accelerated Proximal Gradient approach proposed in [57]. Through the above steps, the low-rank and sparse decomposition process of the weighted matrix has been completed, where the latter contains defect areas.

### 2.4. Pixels Segmentation

After obtaining the sparse matrix, the following features can be observed in combination with Figure 1, Figure 4 and Figure 5.

(1) Imbalanced Data Distribution: From Figure 1, it can be seen that the percentage of small defects in the whole image is very low, which means that the background occupies most of the image, that is, the area difference between defect and defect-free is highly significant.

(2) Zero Pixels Predomination: Most pixels in the sparse matrix are zero (background regions), and the non-zero pixels correspond to defects or noises, as shown in the 3D view of Figure 4 and Figure 5 (the second row). For intuitive visualization, the pixel values of sparse matrix are readjusted to a value of positive integer between 0 and 255, as shown in Figure 4 and Figure 5 (the first row).

After the above analysis, combined with the sparse matrices of Figure 4 and Figure 5, we have two following conclusions. Firstly, the pixel values of the defect are less than 0, and the pixel value greater than 0 is interference such as noise. Secondly, after integer conversion, the mode in images is adopted as the threshold. If pixel values of an area are lower than the mode in images, then the area is regarded as a defect; otherwise, it is regarded as the background. The binarization image is expressed by

(12) $$I_{BW}\left(x,y\right)=\left\{\begin{array}{cc} 1, & I_{S}\left(x,y\right)<mode\left(f\left(x,y\right)\right)\\ 0, & otherwise \end{array}\right.$$

where the defect is represented as 1 and the background is 0.

In order to enhance the consistency of the defect images, for those images containing a small amount of noises or other interference factors, the connected domain pixel value statistics method is used to further instantly remove the interference and accurately segment the defect area.

## 3. Results and Discussions

### 3.1. Experimental Setup

#### 3.1.1. Datasets

To guarantee the effectiveness of the algorithm, we use two publicly available datasets for experimentation. In order to convince the experimental effect, we use the existing methods and the proposed method in this paper for performance evaluation in the same dataset. The dataset used in this experiment is the silicon steel strip dataset (http://faculty.neu.edu.cn/songkechen/zh_CN/zdylm/263273/list/index.htm, accessed: 10 December 2022) [54], which contains a number of color images with a resolution of 480×640×3 pixels. It is characterized by small defect areas, noise interference, complex background, and extremely low contrast between defect and defect-free regions. Another is the magnetic tile dataset (https://github.com/abin24/Magnetic-tile-defect-datasets., accessed: 10 December 2022), consisting of numerous size-varied images. In the experiments, each image is cropped to uniform 270×180 pixels. The contrast between the defect and the background is extremely low in each magnetic tile image, while interfering by stains and textures.

#### 3.1.2. Parameter Settings

The parameters in the implementation of the proposed WMD model are set as follows. In weight matrix construction, to closely match human perception of lightness, we assign weights of the RGB channels based on the CIELAB color space, where $u_{1}$ = 0.212, $u_{2}$ = 0.715, $u_{3}$ = 0.073. In matrix decomposition, λ is related to the number of small defect pixel values, which is set as λ to $1/\sqrt{max(M,N)}$. All experiments are tested on the MATLAB software platform under a machine that is equipped with Intel-i7 2.4-GHz CPU, 16-GB memory.

#### 3.1.3. Comparison Algorithms

The proposed defect detection algorithm is compared with nine state-of-the-art solutions, including six statistical methods (EnOstu [25], VE [23], NVE [26], GVE [24], IVE [27], Ostu [21]), a spectral method (PHOT [38]), and a model-based method (SSD [45]). EnOstu [25] is fully automatic and capable of detecting extremely small defect regions, and the images of low contrast between defect and defect-free. VE [23] is widely used in defect detection, where the applicable defect range can be from no defect to small or large defects. NVE [26] has accurate segmentation results in defect detection, such as a small defect image, a part image, and a number image. GVE [24] resolves such problem of an optimal segmentation threshold selection in a image with unimodal or close to unimodal by introducing a Gaussian weighting scheme to enhance the weighting effect. IVE [27] is suitable for the defect detection with uneven illumination, complex image texture, and relatively small defect area. Ostu [21] is a classical automatic threshold segmentation method. PHOT [38] can be applied to various patterns of defect detection, e.g., periodic texture defect, multiple textures defect of various size and regularities, arbitrary scene with synthetic defect, etc. SSD [45] can detect various types of defects in a smooth background. With the popularization of deep learning methods, a defect classification method based on deep features, called MT [50], is selected as the comparison method of this experiment. In this method, SqueezeNet and MobileNetV2 models are adopted for feature extraction, while the ReliefF algorithm is used for feature selection.

#### 3.1.4. Evaluation Metrics

To evaluate the performance of small surface defect detection methods, we employ nine comparison metrics including accuracy (Ac), sensitivity (Se), specificity (Sp), the Mean Intersection over Union (MIoU), the F-Measure (MF) [58], the Mutual Information (MI), the Normalized Mutual Information (NMI), the Structural Similarity (SSIM) [59], and the E-Measure (EM) [60], respectively. It should be noted that the recall rate and sensitivity are the same. The higher these nine indexes, the better the performance of the algorithm. The common evaluation indicators Ac, Se, Sp, MIoU, MF can be formulated as follows

(13) $$\begin{array}{cc} \; & Ac=\frac{TP+TN}{TP+TN+FP+FN} \end{array}$$

(14) $$\begin{array}{cc} \; & Se=\frac{TP}{TP+FN} \end{array}$$

(15) $$\begin{array}{cc} \; & Sp=\frac{TN}{TN+FP} \end{array}$$

(16) $$\begin{array}{cc} \; & Pr=\frac{TP}{TP+FP} \end{array}$$

(17) $$\begin{array}{cc} \; & MIoU=\frac{TP}{FN+TP+FP} \end{array}$$

(18) $$\begin{array}{cc} \; & MF=\frac{(\beta^{2}+1)\cdot Pr\cdot Se}{\beta^{2}\cdot Pr+Se} \end{array}$$

where TP, TN, FP, and FN represent the number of True Positives, True Negatives, False Positives, and False Negatives, respectively. True Positive (TP) denotes the number of correctly detected defect pixels mapping to the ground truth. True Negatives (TN) represents the number of correctly detected background pixels mapping to the ground truth. False Positive (FP) is the number of defect pixels that are false detected. False Negative (FN) represents the number of undetected defect pixels. In general, the improvement of precision corresponds to the decrease of recall. For the MF metric, we use $\beta^{2}=1$ in this paper to balance precision and recall.

We also compare the detected result with ground truth by calculating their pixel and structure similarity. Specifically, MI and NMI metrics compute pixels similarity between the test image and the ground truth, while the SSIM and EM compute the structural similarity.

(19) $$SSIM=\frac{\left(2u_{x}u_{y}+c_{1}\right)\left(2\delta_{xy}+c_{2}\right)}{\left(u_{x}^{2}+u_{y}^{2}+c_{1}\right)\left(\delta_{x}^{2}+\delta_{y}^{2}+c_{2}\right)}$$

where $u_{x}$ and $u_{y}$ are the mean values of *x* and *y*, respectively. $\delta_{x}^{2}$ and $\delta_{y}^{2}$ are the variances of *x* and *y*, respectively. $\delta_{xy}$ is the covariance of *x* and *y*. $c_{1}$ and $c_{2}$ are constants. EM comprehensively evaluates image quality from pixel, region, boundary, and object level [60], that is

(20) $$EM=\frac{1}{w+h}\sum_{x=1}\sum_{y=1}\phi_{FM}\left(x,y\right)$$

where *w* and *h* are the width and height of image, respectively. $\phi_{FM}$ is an enhanced alignment matrix.

### 3.2. Comparison With the State-of-the-Art

**Figure 7:**
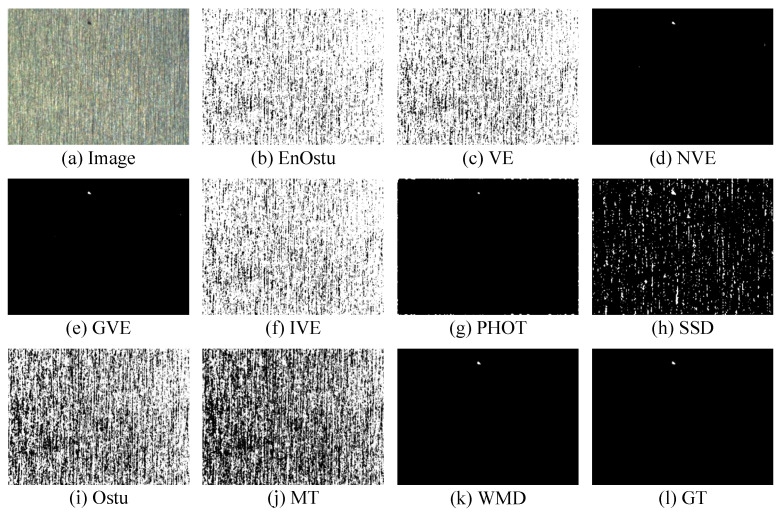
Visual comparisons of detection results of the ten methods on the image with a single defect. Our detection result (WMD) is quite close to ground truth.

**Figure 8:**
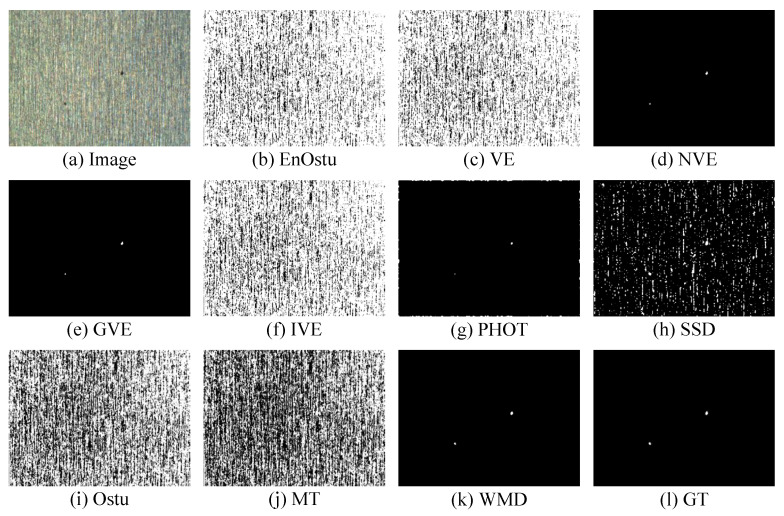
Visual comparisons of detection results of the ten methods on the image with multiple defects. The detection result of the proposed WMD method is quite close to ground truth, whereas the other competing methods are unable to either identify the defect location or completely separate the defect from the background.

**Figure 9:**
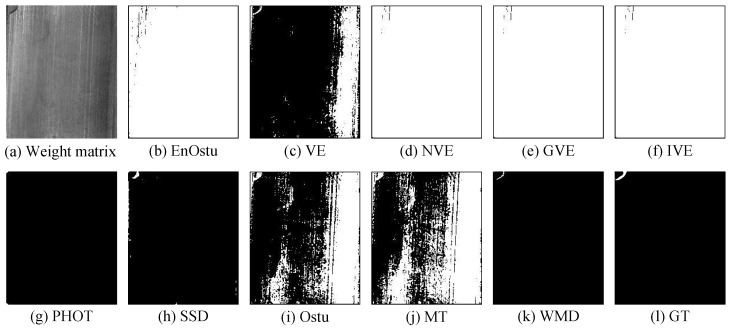
Visual comparisons of detection results of the ten methods on the image with multiple defects. The detection result of the proposed WMD method is quite close to ground truth, whereas the other competing methods are unable to either identify the defect location or completely separate the defect from the background.

**Figure 10:**
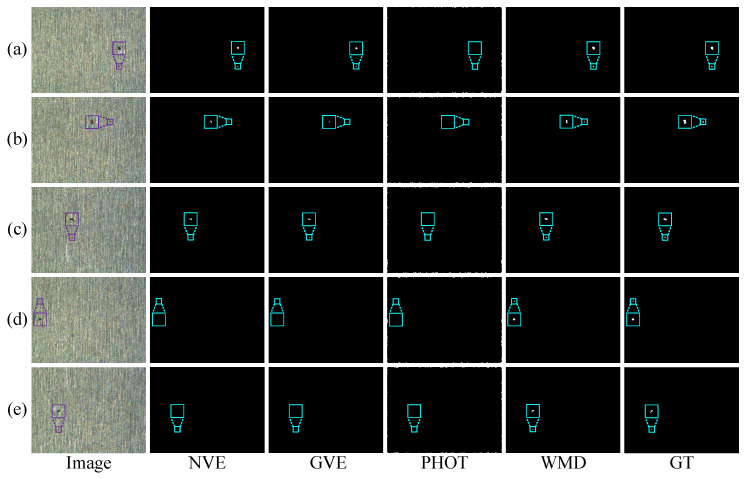
Visual comparisons of four methods on (**a**–**e**) five randomly selected single-defect images. The detection result of the proposed WMD method is quite close to ground truth (GT), whereas the other competing methods are unable to either identify the defect location or completely separate the defect from the background.

**Figure 11:**
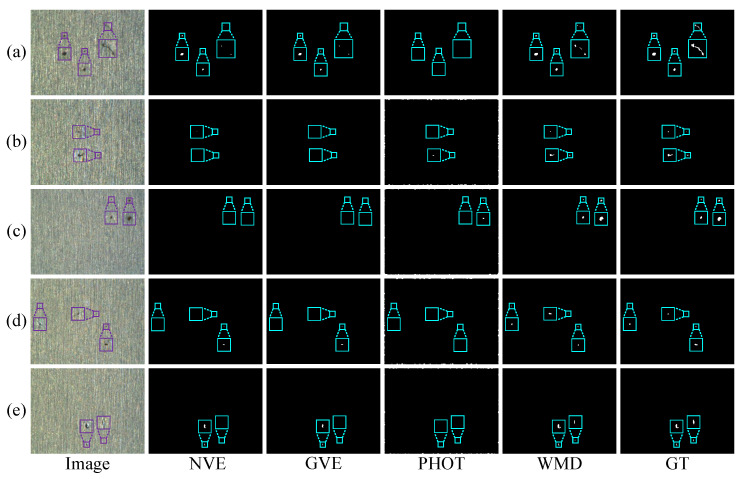
Visual comparisons of four methods on (**a**–**e**) five randomly selected multiple-defect images. The detection result of the proposed WMD method is quite close to ground truth (GT), whereas the other competing methods are unable to either identify the defect location or completely separate the defect from the background.

**Figure 12:**
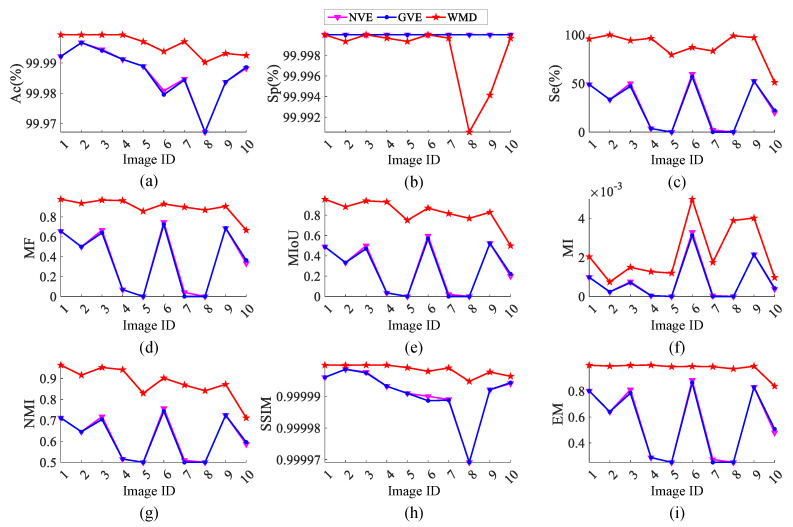
Quantitative comparison in terms of (a) Ac(%), (**b**) Sp(%), (**c**) Se(%), (**d**) MF, (**e**) MIoU, (**f**) MI, (**g**) NMI, (**h**) SSIM, and (**i**) EM, where the *x* axis is the Image ID. Our method (WMD) significantly outperforms these evaluated defect detection algorithms across ten randomly selected surface defect detection images.

The proposed WMD algorithm is evaluated on the small surface defect detection dataset and compared with nine recently proposed algorithms. The visual comparisons are shown in Figure 7, Figure 8, Figure 9, Figure 10, Figure 11 and Figure 12, and Table 1 shows some quantitative comparison. The results show that the proposed WMD, an unsupervised method, ranks first on the small surface defect detection dataset across different criteria.

#### 3.2.1. Visual Comparison

Figure 7, Figure 8 and Figure 9 show the visualization of partial detection results. In Figure 7 and Figure 8, we can observe that EnOstu [25], VE [23], IVE [27], SSD [45], Ostu [21], and MT [50] cannot identify the location of the defect. NVE [26], GVE [24], PHOT [38], and our method (WMD) are able to effectively separate the defect from the background. In Figure 9, EnOstu [25], VE [23], NVE [26], GVE [24], and IVE [27] almost judge the background as a defect. PHOT [38] and SSD [45] identify background and a small number of defects. Ostu [21] and MT [50] are powerless against magnetic tile defects.

To further evaluate the performance of these methods, we conduct two experiments on five randomly selected single-defect images and multiple-defect images in a silicon steel strip dataset, as shown in Figure 10 and Figure 11, respectively. In these two experiments, the results from EnOstu [25], VE [23], IVE [27], SSD [45], Ostu [21], and MT [50] methods are not present because they are incapable of detecting defects and their results contain a number of false positives. Similarly, there is no experimental result for the magnetic tile image because the traditional method is similar to Figure 9 in other images. In Figure 10, PHOT [38] is unable to effectively identify defects on all the five images. The NVE [26] and GVE [24] methods can detect relatively salient defects in (a)–(c) and partial defect regions in (d), but fail to detect the defect that is highly similar to the background in (e). The method proposed (WMD) in this paper achieves the best detection results in the above five images. In Figure 11, we obtain a conclusion similar to that in Figure 10.

#### 3.2.2. Performance Comparison

It can be seen that the methods of NVE [26] and GVE [24] are suitable for the detection of small surface defects by the comparison of the above experiments. Figure 12 shows some quantitative comparisons by the three methods (NVE, GVE, and WMD). As shown in Figure 12a,c–i, Ac(%), Se(%), MF, MIoU, MI, NMI, SSIM, and EM are clearly higher than those of the baseline methods. Sp represents the probability of detected background pixels, and Se denotes the probability of detected defect pixels. However, it should be noted that recall is more indispensable than precision in small surface defect detection, in respect that a missed defect has a greater hazard than an error-checked noise. The performance comparison demonstrates that the proposed WMD model performs better than all the competitors in detecting the images with single defect and multiple defects.

To quantify the effect of Figure 9, the evaluation indicators of ten methods are shown in Table 1. As can be seen, the WMD works best in the Ac(%), Pr(%), Se(%), MF, MIoU, NMI, SSIM, and EM indicators. The Sp value of EnOstu [25], VE [23], NVE [26], GVE [24], and IVE [27] respectively reaches 100%, because the five methods almost all judge the background as defects. However, their Pr and Sp values are extremely low, and the Ac value for correct identification of background and defects is also quite small. The above results demonstrate the effectiveness of the proposed method in the magnetic tile image.

### 3.3. Verify the Effectiveness of Weighted Matrix Decomposition

**Figure 13:**
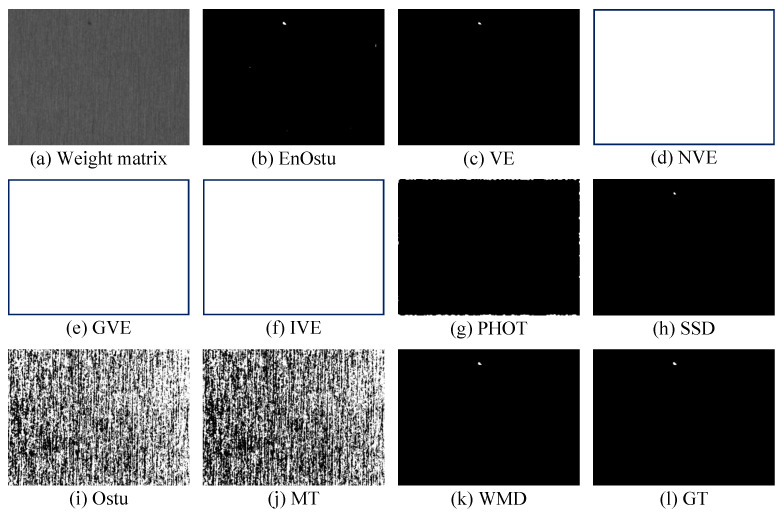
Visual comparisons of detection results using the ten methods on weighed matrix. NVE, GVE, IVE, and PHOT do not have any output. EnOstu, VE, SSD, Ostu, MT, and our method (WMD) have the ability to separate defects from the background.

**Figure 14:**
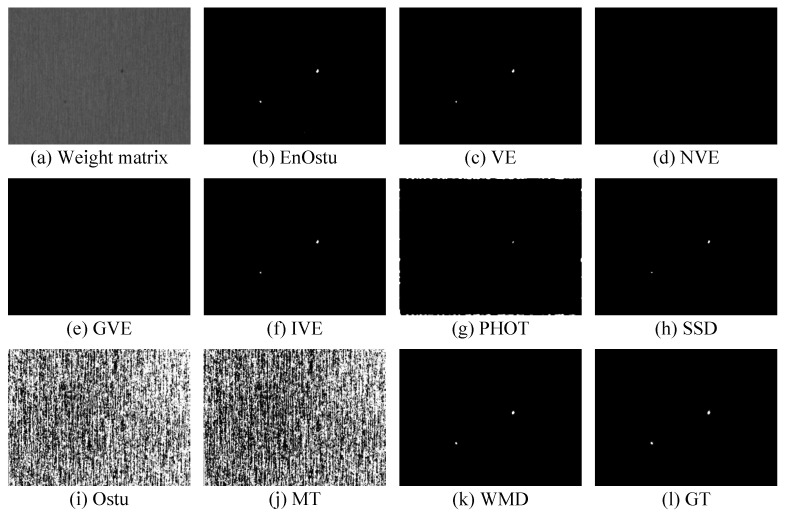
Visual comparisons of detection results using the ten methods on weighed matrix with multiple defects. NVE, GVE, and PHOT cannot effectively identify defects. EnOstu, VE, IVE, SSD, Ostu, MT and our method (WMD) can effectively provide the location of the defect.

**Figure 15:**
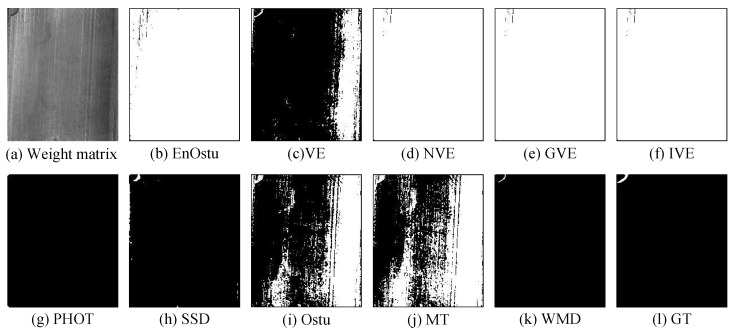
Visual comparisons of detection results using the ten methods on weighed matrix of magnetic tile images. EnOstu, VE, NVE, GVE, IVE, PHOT, SSD, Ostu, and MT cannot effectively identify defects. VE only identifies some defects, and our method (WMD) can effectively provide the location of defect.

**Figure 16:**
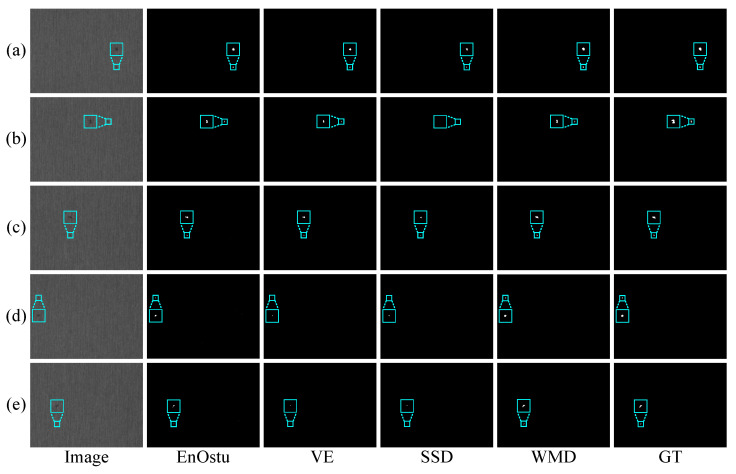
Visual comparisons of different methods on (**a**–**e**) five randomly selected single-defect images. The ground truth (GT) is shown in the last column. EnOstu, VE, SSD, and our method (WMD) have the ability to locate the defect.

**Figure 17:**
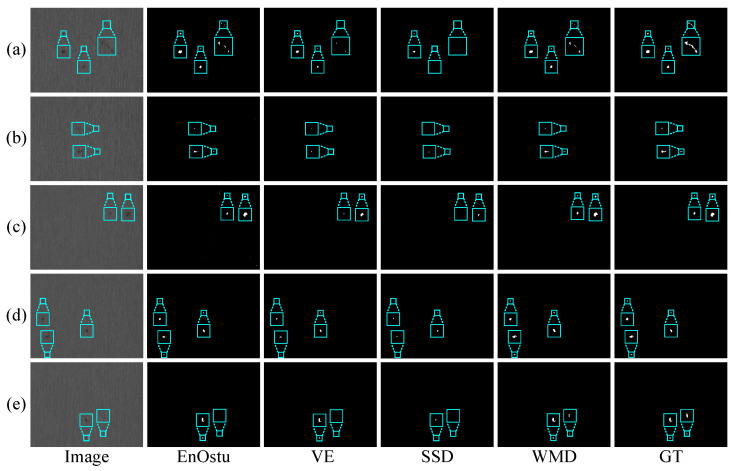
Visual comparisons of different methods on (**a**–**e**) five randomly selected multiple-defect images. Compared with the baseline methods, the proposed WMD produces more discriminative models, which is capable of identifying the defect from backgrounds.

**Figure 18:**
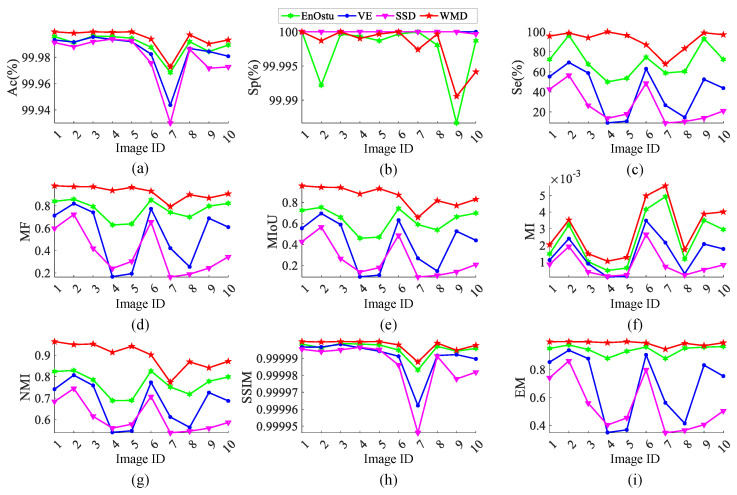
Quantitative comparison in terms of (**a**) Ac(%), (**b**) Sp(%), (**c**) Se(%), (**d**) MF, (**e**) MIoU, (**f**) MI, (**g**) NMI, (**h**) SSIM, and (**i**) EM, where the *x* axis is the Image ID. Our method outperforms the baseline methods.

In the experiment comparison in Section 3.2, the small surface defect images are converted to gray images, then the existing methods are used to perform defect detection. However, in Section 2.2, the contrast between the defect and the background of the weighted matrix is greater than that in the gray images (Figure 3). To verify the effectiveness of low-rank and sparse matrices decomposition in defect detection, all methods are simultaneously used in the weighted matrix. Visual comparisons are shown in Figure 13, Figure 14, Figure 15, Figure 16 and Figure 17, while some quantitative comparisons are presented in Figure 18 and Table 2.

#### 3.3.1. Visual Effect Verification

Figure 13, Figure 14 and Figure 15 show the visualization results. As can be seen, the detection effect of NVE [26], GVE [24], and IVE [27] present great distinction between single-defect and multiple-defect images. In Figure 13, these methods cannot identify background and defects, whereas in Figure 14, they fail to separate the defect or only part of the region. Similar conclusions can be obtained in other small surface defect images. Ostu [21] and MT [50] are unable to distinguish defects and backgrounds in Figure 13 and Figure 14. PHOT [38] only identifies one of the defects in a small number of multi-defect images, and performs poorly in single-defect images. The results are similar with those in Section 3.2. EnOstu [25], VE [23], SSD [45], and our method (WMD) are capable of discriminating the defect regions. In Figure 15, the visual results of the EnOstu [25], NVE [26], GVE [24], IVE [27], PHOT [38], SSD [45], Ostu [21], and MT [50] in the weighted matrix are similar to those in Figure 9. The detection performance of the VE [23] in the weighted matrix is better than that in the original image, but it only detects part of the defects, and also mistakenly judges some background as defects. Our method (WMD) is capable of separating defects from the background.

Figure 16 and Figure 17 show some visual comparisons of the best methods in the experiments. As can be seen from Figure 16, EnOstu [25], VE [23], and WMD successfully separate the defects from the background. However, SSD [45] occasionally fails to identify the defect. In Figure 17a–d, EnOstu [25] and VE [23] correctly detect small defects, but are incapable of detecting defects similar to the background in Figure 17e. SSD [45] is unable to detect the defects as small as a few pixels or similar to the background. The method proposed (WMD) in this paper successfully locates the defects in the above five images.

#### 3.3.2. Performance Metrics Verification

Figure 18 shows some quantitative comparison of the best methods (EnOstu [25], VE [23], SSD [45], and WMD). It can be concluded that the proposed model WMD in this paper has the best performance in terms of eight evaluation metrics (Figure 18a,c–i). The quantitative evaluation results in Figure 15 are shown in Table 2, which yields similar conclusions to Table 1. Among them, the Pr and Sp values of PHOT [38] are both 0, indicating that it judges all defects as background. However, the AC value reaches 99.77691%, because the proportion of defects in the image is extremely low, and the background is particularly large. The quantitative evaluation results in Figure 15 are shown in Table 2, which yields similar conclusions to Table 1. Among them, the Pr and Sp values of PHOT [38] are both 0, indicating that it judges all defects as background. However, the AC value reaches 99.77691%, because the proportion of defects in the image is quite low, and the background is particularly large.

In summary, for the images whose defect and defect-free region share similar appearance, WMD successfully separates the defects from the background, while other methods often fail. These results illustrate the robustness of the WMD algorithm, and confirm the effectiveness of the proposed weighted matrix in separating the low-rank and sparse subspaces.

## 4. Conclusions

In this paper, we have presented a weighted matrix decomposition model which effectively solves the problem of detecting small surface defects. Firstly, a weighted matrix is established to improve the contrast between small defects and background. Then, the weighted matrix is decomposed into a low-rank matrix representing image background and a sparse matrix identifying defects, which eliminates the interference of complex backgrounds. Finally, the pixel intensity of the defects is analyzed to select the optimal threshold and accurately separate the defects from the background. Experiments on a small surface defect dataset have shown that, compared to the conventional methods, the proposed achieves encouraging performance in small defect detection against complex product surfaces on public benchmark datasets under various quantitative metrics. The proposed model provides an effective solution in industrial manufacturing for detecting small defects against complex product surfaces. In the future, we will further generalize our 2D patch model into 3D or more dimensions and investigate applications of the N-D patch model. We will also try multi-subspace cluster strategies to further improve the flexibility of our method in highly variant background cases.

## Figures and Tables

**Table 1:** Results on the magnetic tile image in terms of Ac(%), Pr(%), Sp(%), Se(%), MF, MIoU, MI, NMI, SSIM, and EM.

Methods	Ac(%)	Pr(%)	Sp(%)	Se(%)	MF	MIoU	NMI	SSIM	EM
EnOtsu	0.45013	0.19990	**100**	0.25123	0.00399	0.00200	0.50008	0.86746	0.00130
VE	10.49514	0.22229	**100**	10.31631	0.00444	0.00222	0.50031	0.88316	0.23347
NVE	0.27837	0.19956	**100**	0.07913	0.00398	0.00200	0.50004	0.86711	0.02549
GVE	0.27837	0.19956	**100**	0.07913	0.00398	0.00200	0.50004	0.86711	0.02549
IVE	0.27837	0.19956	**100**	0.07913	0.00398	0.00200	0.50004	0.86711	0.02549
PHOT	99.66240	8.33333	6.93069	99.84768	0.07568	0.03933	0.50745	0.99984	0.58086
SSD	99.84009	79.41176	26.73267	99.98615	0.40000	0.25000	0.59021	0.99992	0.64337
Ostu	36.86725	0.30245	96.03960	36.74903	0.00603	0.00302	0.50046	0.92478	0.24855
MT	50.75417	0.38346	95.04950	50.66566	0.00764	0.00383	0.50072	0.94407	0.25117
WMD	**99.86378**	**94.44444**	33.66337	**99.99604**	**0.49635**	**0.33010**	**0.62883**	**0.99994**	**0.66594**

**Table 2:** Results on weight matrix of magnetic tile image in terms of Ac(%), Pr(%), Sp(%), Se(%), MF, MIoU, MI, NMI, SSIM, and EM.

Methods	Ac(%)	Pr(%)	Sp(%)	Se(%)	MF	MIoU	NMI	SSIM	EM
EnOtsu	0.55279	0.20011	**100**	0.35410	0.00399	0.00200	0.50009	0.86766	0.00655
VE	80.78457	0.63551	61.38614	80.82333	0.01258	0.00633	0.50085	0.97779	0.25874
NVE	0.28627	0.19957	**100**	0.08704	0.00398	0.00200	0.50004	0.86712	0.01858
GVE	0.28627	0.19957	**100**	0.08704	0.00398	0.00200	0.50004	0.86712	0.01858
IVE	0.28627	0.19957	**100**	0.08704	0.00398	0.00200	0.50004	0.86712	0.01858
PHOT	99.77691	0	0	99.97626	0	0	0.50001	0.99987	0.38060
SSD	99.60317	27.67857	61.38614	99.67953	0.38154	0.23574	0.57370	0.99986	0.66337
Ostu	62.84056	0.48664	91.08911	62.78412	0.00968	0.00486	0.50095	0.95823	0.25334
MT	49.19450	0.36789	94.05941	49.10486	0.00733	0.00368	0.50064	0.94140	0.25088
WMD	**99.86378**	**94.44444**	33.66337	**99.99604**	**0.49635**	**0.33010**	**0.62883**	**0.99994**	**0.66594**

## Data Availability

Not applicable.
